# The Consequences of Multiple Simultaneous C-Type Lectin–Ligand Interactions: DCIR Alters the Endo-Lysosomal Routing of DC-SIGN

**DOI:** 10.3389/fimmu.2015.00087

**Published:** 2015-03-10

**Authors:** Juan J. García-Vallejo, Karien Bloem, Léon M. J. Knippels, Johan Garssen, Sandra J. van Vliet, Yvette van Kooyk

**Affiliations:** ^1^Department of Molecular Cell Biology and Immunology, VU University Medical Center, Amsterdam, Netherlands; ^2^Danone Research, Centre for Specialized Nutrition, Wageningen, Netherlands; ^3^Department of Pharmacology and Pathophysiology, Utrecht Institute for Pharmaceutical Sciences (UIPS), Utrecht University, Utrecht, Netherlands

**Keywords:** mannose receptor, dendritic cell-specific intercellular adhesion molecule-3-grabbing non-integrin, dendritic cell immunoreceptor, macrophage galactose-type lectin, dendritic cells, antigen uptake, intracellular routing

## Abstract

Antigen-presenting cells (APCs) are equipped with multiple receptors to allow proper pathogen recognition and capture. C-type lectin receptors (CLRs) recognize glycan structures on pathogens and endogenous glycoproteins for internalization and antigen processing and presentation. Often, the glycan specificity of these receptors is overlapping and/or pathogens are decorated with ligands for multiple CLRs, posing the question whether interference or cooperativity within the CLR family exists. Here, we used imaging flow cytometry to investigate the internalization properties of four different CLRs [mannose receptor, DC-specific intercellular adhesion molecule-3-grabbing non-integrin (DC-SIGN), macrophage galactose-type lectin, and dendritic cell immunoreceptor (DCIR)] on different APCs, as well as their intracellular routing. Although the internalization score of the investigated CLRs was similar on monocytes, macrophages, and dendritic cells (DCs), DCIR internalization rates were lower compared to the other CLRs. Upon triggering, DCIR routed to intracellular compartments outside of the classical endo-lysosomal pathway, resulting in poor CD4^+^ T-cell stimulation. Although DC maturation reduced CLR expression levels, it did not affect their internalization rates. Although CLR internalization appeared to be independently regulated, DC-SIGN routing was affected when DCIR was triggered simultaneously. In conclusion, our results provide new insights for the design of DC-based immunotherapeutic strategies and suggest that DCIR is an inferior target in this respect.

## Introduction

Antigen-presenting cells (APCs) play a pivotal role in the activation of T cells and the organization of the immune response. Amongst APCs, dendritic cells (DCs) have the highest expression of major histocompatibility complex (MHC) molecules to allow for antigen presentation (1–3). In addition, DCs are equipped with a broad set of receptors, such as toll-like receptors (TLRs) that recognize different pathogen-associated molecular patterns (PAMPs) (4). Upon recognition, signaling events result in DC maturation, a phenomenon characterized by the up-regulation of co-stimulatory molecules and the secretion of cytokines to provide the necessary signals that activate T cells (5).

Other receptors, including C-type lectin receptors (CLRs), facilitate antigen uptake and mediate the routing of the internalized antigens to MHC-I- or MHC-II-loading compartments for their presentation to T cells (6). CLR signaling has also been shown to modulate TLR responses (7, 8). CLRs mediate the recognition of glycan structures on both pathogens and endogenous proteins (9). The intracellular route followed by internalized antigens depends on the mechanism of internalization (10). Most commonly, after receptor-mediated endocytosis, internalized antigens enter the endo-lysosomal route, where degradation of the antigen is initiated. Antigen-derived peptides are loaded onto MHC-II molecules, which travel to the cell membrane for CD4^+^ T-cell stimulation (2). DC-mediated cross-presentation occurs predominantly via the cytosolic pathway or the vacuolar pathway (3), leading to peptide presentation in MHC-I and activation of CD8^+^ T cells. Other APCs, such as monocytes and macrophages also express CLRs and TLRs; however, they have an inferior capacity to stimulate T cells as compared to DCs (1, 2).

Antigen internalization and routing to MHC-loading compartments has been reported for various CLRs. The mannose receptor (MR) routes its ligands to endo-lysosomes (11), resulting in T-cell proliferation (12, 13). Increased antigen uptake is achieved by recycling of MR from intracellular pools (14, 15). In contrast, DC-specific intercellular adhesion molecule-3-grabbing non-integrin (DC-SIGN) is exclusively expressed on the plasma membrane (16). DC-SIGN ligands are quickly internalized and routed to endosomes and lysosomes (16–20), predicting MHC-II presentation. Indeed, DC-SIGN-internalized antigens activate CD4^+^ T-cell proliferation, however stimulation of CD8^+^ T-cell responses has also been observed for DC-SIGN-binding ligands (1–6). Similar endosomal targeting and CD4^+^ T-cell activation has been reported for ligands of the human macrophage galactose-type lectin (MGL) (1, 3, 5, 7). Although routing to MHC-I compartments has been described (8, 9), stimulation of CD8^+^ T-cell responses has not been demonstrated for human MGL yet. Dendritic cell immunoreceptor (DCIR) targets antigens for both CD4^+^ and CD8^+^ T-cell presentation (10–12), although only the routing to lysosomes has been formally demonstrated (13, 14). Compared to other CLRS, DCIR internalization seems to occur less efficiently (15–17). It is currently unknown whether DCIR and MGL are able to recycle to the membrane from intracellular pools.

Due to their overlapping ligand specificity, glycosylated antigens may interact with multiple CLRs simultaneously. For example, MR, DCIR, and DC-SIGN interact with mannose and fucose-rich glycans and share recognition of certain pathogens, including HIV-1 (18–21). In addition, pathogens may carry multiple glycan determinants that facilitate recognition by different CLRs. Even though MGL and DC-SIGN bind distinct glycan epitopes, they both recognize soluble egg antigens from the pathogen *Schistosoma mansoni* (22, 23).

We therefore investigated by imaging flow cytometry the internalization of MR, DC-SIGN, MGL, and DCIR in different mature and immature APCs after single or simultaneous triggering of the CLRs. We here report a distinct role for DCIR in antigen presentation, a dissimilar antigen uptake capacity of DCIR and MGL on different APCs and interference of DCIR triggering on DC-SIGN routing.

## Materials and Methods

### Reagents and antibodies

The following reagents were used: paraformaldehyde (PFA; formaldehyde) aqueous solution (Electron Microscopy Sciences; PFA), saponin (Sigma-Aldrich), *Escherichia coli* lipopolysaccharide (LPS; 0111; B4, Sigma-Aldrich), and bovine serum albumin (BSA; Roche). The following antibodies were used: α-DC-SIGN (clone AZN-D1) (3, 24), α-DCIR (clone 111F8.04, unlabeled, Alexa Fluor (AF) 488 and 647 labeled, Dendritics), α-MR (clone 19.2, BD Bioscience), α-MGL (clone 125A10.03, unlabeled and AF647 labeled, Dendritics), α-ICAM2 [12A2 (5, 25) EEA-1-FITC, clone 14/EEA-1, BD Biosciences], HLA-DM-PE (clone MaP.DM1, BD Biosciences), LAMP-FITC (clone H4A3, BD Biosciences), rab 5 (clone FL-215, Santa Cruz Biotechnology), rab 7 (clone H-50, Santa Cruz Biotechnology), PDI-PE (clone 1D3, Assay designs), TGN46 (ab56726, Abcam), polyclonal rabbit-α-rab 11 (Invitrogen), Pacific orange-labeled goat-α-rabbit IgG (Invitrogen), AF 594-labeled goat-α-mouse IgG_2a_ (Invitrogen), IFNγ coating and biotin-labeled IFNγ detection antibody (Invitrogen). α-DC-SIGN AZN-D1 was labeled with AF405, α-MGL 125A10.03 with AF594, and α-MR 19.2 with AF647 (Invitrogen) according to manufacturer’s instructions.

### Cells

Monocytes were isolated from peripheral blood mononuclear cells (PBMCs) from buffy coats of healthy donors (Sanquin) by a lymphoprep gradient (Axis-Shield) and subsequent percoll gradient centrifugation (Amersham). Informed consent was obtained from all blood donors for the use of their blood. DCs were generated by culturing purified monocytes in RPMI1640 (Invitrogen) supplemented with 10% fetal bovine serum (BioWhittaker), 1000 U/ml penicillin/streptomycin (Lonza), and 2 mM glutamine (Lonza) in combination with IL-4 (262.5 U/ml; Biosource) and GM-CSF (112.5 U/ml; Biosource) for 4–7 days. Ten nanograms per milliliter LPS was added for indicated time periods to mature cells. HD7 cells, a CD4^+^ T-cell clone that recognizes a peptide derived from mouse IgG_1_ antibodies in HLA-DR0101/DQw1, were used as T-cell responders (1, 3, 26).

### Intracellular CLR and subcellular compartment staining

Cells were washed in ice-cold phosphate-buffered saline (PBS), fixed in ice-cold 4% PFA in PBS for 20 min, and then washed two times with ice-cold PBS. For intracellular stainings, cells were permeabilized in 0.1% saponin in PBS for 30 min at room temperature and then blocked with a solution containing 0.1% saponin, 2% BSA, and 1% goat serum in PBS. Primary and secondary stainings were performed in PBS supplemented with 0.1% saponin and 2% BSA at room temperature. After staining, cells were kept at 4°C in PBS supplemented with 0.05% BSA and 0.02% sodium azide until analysis.

### Internalization, recycling, and combined routing experiments

Cells (1 million) were incubated for 20 min in 100 μl of ice-cold RPMI 1640 medium (Invitrogen) containing 10% fetal bovine serum. α-DC-SIGN, α-DCIR, α-MGL, and/or α-MR were added and incubated for 30 min on ice to allow binding to cell surface CLRs without triggering internalization. Cells were then transferred to 37°C for 1 h or kept on ice. Cells were washed in ice-cold PBS, fixed in ice-cold 4% PFA in PBS for 20 min, and then washed two times in ice-cold PBS. Cells were kept at 4°C in PBS supplemented with 0.05% BSA and 0.02% sodium azide until analysis.

### Intracellular routing experiments

Cells (1 million) were incubated for 20 min in 100 μl of ice-cold RPMI 1640 medium (Invitrogen) containing 10% fetal bovine serum. α-DC-SIGN and/or α-DCIR were added and incubated for 30 min on ice to allow binding to cell surface DC-SIGN and/or DCIR without triggering internalization. Cells were washed in ice-cold medium to remove unbound antibodies and then transferred to 37°C for different time-points or kept on ice. At the desired time-points, cells were washed in ice-cold PBS, fixed in ice-cold 4% PFA in PBS, and stained for markers of intracellular routing.

### Imaging flow cytometry

**Figure 1:**
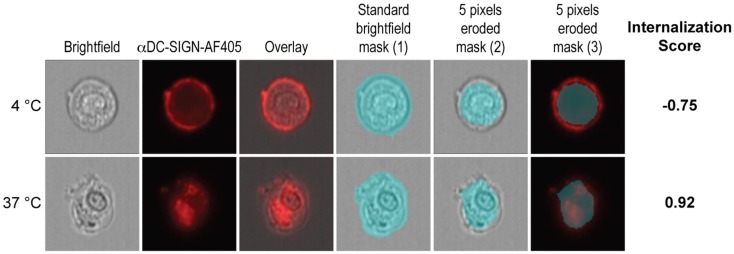
**Calculation of the internalization score**. Firstly, the *morphology* mask was applied to brightfield images (1). Then, 5 pixels were evenly eroded from the border of the mask in order to exclude the cell membrane from the mask (2). The resulting mask was applied to the fluorescence channel (3). The internalization feature was then applied to the final mask (3) in order to calculate the internalization score.

Cells were analyzed on the ImageStreamX (Amnis Corp.) imaging flow cytometer as previously described (4, 27). A minimum of 15,000 cells were acquired per sample. Internalization and co-localization scores were calculated as previously described (28–30). Briefly, cells were acquired on the basis of their area. Analysis was performed with single cells after compensation (with a minimum of 5000 cells). Internalization scores were calculated as described in Figure 1. Firstly, a mask was designed based on the surface of DCs in the brightfield image (1). This mask was then eroded to exclude the cell membrane (2). Finally, the resulting mask was applied to the fluorescence channel (3). The internalization score was then calculated on this mask using the *Internalization* feature provided in the Ideas v6.0 software (Amnis Corp.). Internalization can be interpreted as a log-scaled ratio of the intensity of the intracellular space versus the intensity of the entire cell. Cells that have internalized antigen typically have positive scores, while cells that show the antigen still on the membrane have negative scores. Cells with scores around 0 have similar amounts of antigen on the membrane and in intracellular compartments. Co-localization is calculated using the *bright detail similarity R3* feature in the Ideas software. This feature corresponds to the logarithmic transformation of Pearson’s correlation coefficient of the localized bright spots with a radius of 3 pixels or less within the whole cell area in the two input images.

### T-cell stimulation experiments

20 × 10^3^ DCs/well were pre-incubated with serial dilutions of α-DC-SIGN, α-DCIR, α-MR, α-MGL or isotype control antibody for 2 h at 37°C and subsequently co-cultured with 80 × 10^3^ HD7 cells for 48 h. Afterwards, IFNγ production in the supernatant was measured by ELISA. Shortly, IFNγ capture antibody was coated in 50 mM NaHCO_3_, pH 9.7. Plates were blocked with 1% BSA in PBS. Diluted supernatants and IFNγ detection antibody were added and presence of IFNγ was detected with streptavidin-PO (Invitrogen). Binding was visualized with 3,3′,5,5′-tetramethylbenzidine (Sigma-Aldrich) as substrate and optical density was measured by spectrophotometry at 450 nm.

## Results

### MGL and DCIR are recycling receptors in human DCs

**Figure 2:**
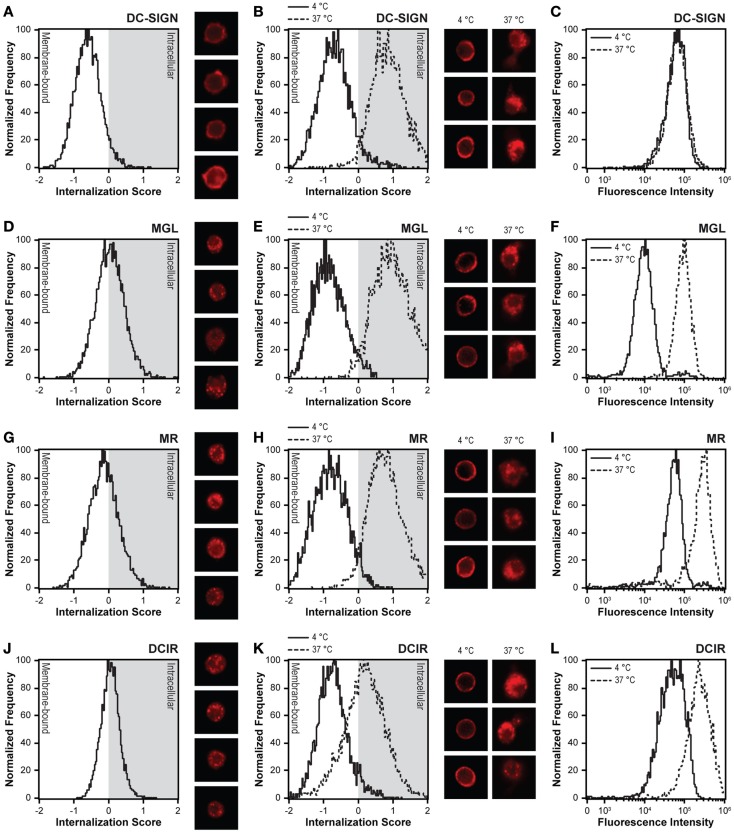
**DC-SIGN is the only CLR expressed exclusively on the cell membrane and lacking recycling capacity**. **(A,D,G,J)** Distribution of CLRs was investigated by intracellular staining of DCs with CLR-specific monoclonal antibodies. Negative internalization scores indicate membrane expression and positive internalization scores represent intracellular staining. Four representative images are displayed next to the internalization scores. **(B,E,H,K)** CLR internalization was investigated by allowing internalization of membrane-expressed CLRs for 1 h. Staining at 4°C represents membrane-bound CLRs, whereas staining at 37°C indicates internalized CLRs. Three representative images of both conditions are displayed next to the internalization scores. **(C,F,I,L)** The recycling capacity was investigated by allowing CLR internalization in the presence of excess amount of antibody. CLR-specific antibodies were allowed to bind membrane-expressed CLRs at 4°C. CLR internalization was initiated through incubation at 37°C. Recycling of receptors to the membrane is indicated by an increased fluorescent intensity at 37°C compared to membrane staining at 4°C. Results are representative of three independent experiments.

To elucidate differences in antigen internalization and routing by MR, DC-SIGN, MGL, and DCIR, we first compared the distribution of these CLRs on DCs. Resting DCs were fixed, permeabilized, stained for each of the abovementioned CLRs, and measured by imaging flow cytometry. Localization of the CLR on the cell membrane or in intracellular compartments was addressed using the internalization feature. Internalization scores below 0 indicate that the CLR is located on the plasma membrane, while positive internalization scores point to intracellular expression. Consequently, internalization scores close to 0 indicate an equal distribution between membrane and intracellular compartments. As previously demonstrated, DC-SIGN was exclusively localized at the cell membrane of DCs, while MR was distributed mainly in intracellular compartments (28, 31, 32). The CLRs MGL and DCIR showed a cellular distribution similar to the MR, with approximately half of the molecules located on the cell membrane and the other half intracellularly (Figures 2A,D,G,J).

Internalization was measured according to classical pulse-chase experiments, by first incubating DCs with the anti-CLR antibodies at 4°C to allow binding of the antibodies to the receptor, followed by a washing step to remove unbound antibodies and a final incubation at 37°C for 60 min. As expected, all anti-CLR antibodies showed an exclusive cell membrane localization at 4°C, which were efficiently internalized upon incubation at 37°C. Compared to the other CLRs and in agreement with previous research, the internalization score of DCIR was lower, indicating that more DCIR molecules remain on the cell membrane after receptor internalization (Figures 2B,E,H,K) (14, 33).

Since MGL, MR, and DCIR were located both at the cell membrane and in intracellular compartments, we speculated that this might be explained by continuous receptor recycling allowing for accumulated antigen uptake, as has already been observed for MR (4, 31–36). Therefore, DCs were incubated with the anti-CLR antibodies at 4°C for 30 min and directly transferred to 37°C, without removal of unbound antibodies. Indeed, compared to exclusive membrane staining at 4°C, MGL, MR, and DCIR showed an increased staining at 37°C, when receptor recycling and an accumulation of staining is possible. In contrast, DC-SIGN displayed comparable staining at 4 and 37°C (Figures 2C,F,I,L), indicating an absence of DC-SIGN recycling to the plasma membrane during the time frame of the experiment, in line with previous reports (2, 4, 6, 37).

### DC maturation does not alter the internalization capacity of CLRs

**Figure 3:**
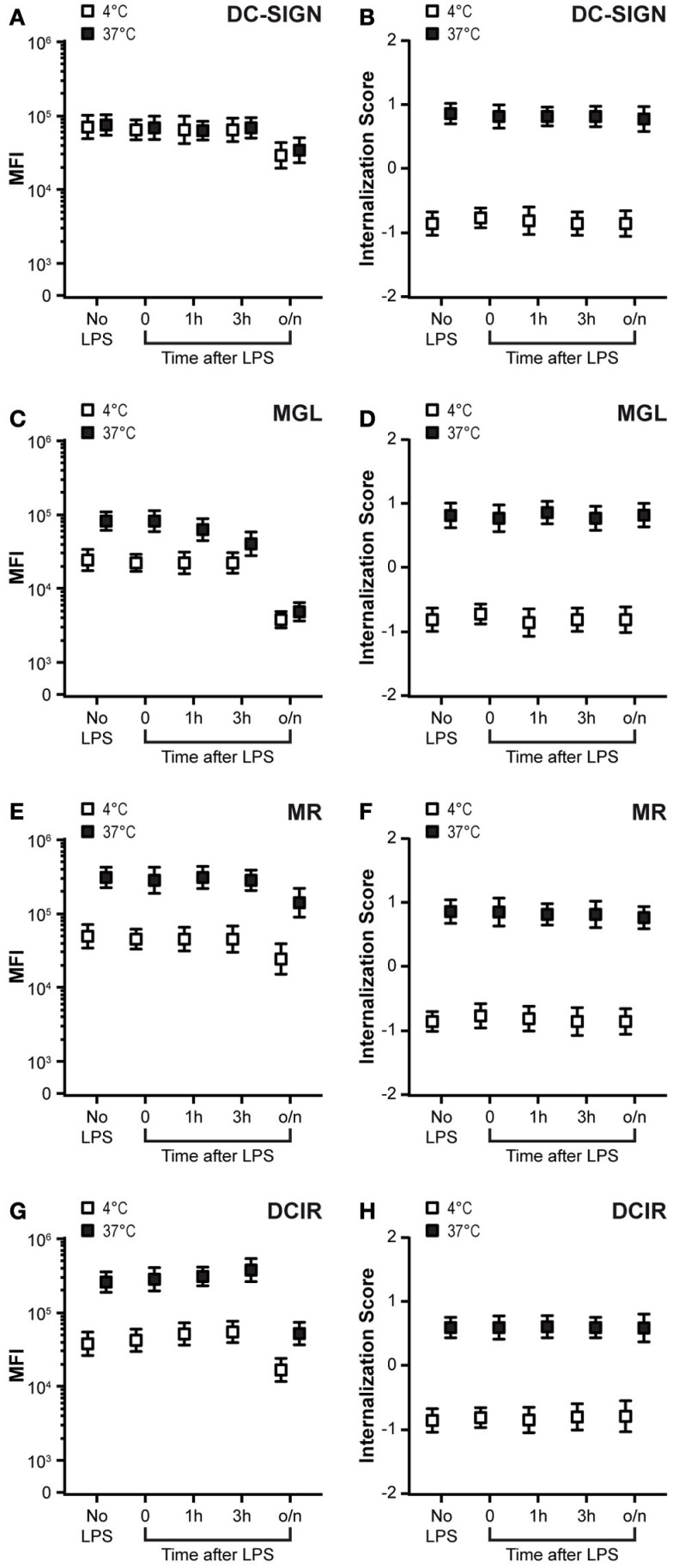
**DC maturation diminishes MGL recycling**. **(A,C,E,G)** DCs were exposed to LPS (10 ng/ml) for the indicated time-points. Membrane-expressed CLRs were stained at 4°C with CLR-specific antibodies (white squares) and directly transferred (without washing) to 37°C allowing CLR internalization and recycling (black squares). Staining is depicted as the median fluorescent intensity (MFI) ± SE of a minimum of 5000 events. **(B,D,F,H)** Internalization scores of CLRs after addition of LPS for indicated time-points. Membrane-expressed CLRs were stained at 4°C with CLR-specific antibodies (white squares), washed, and incubated for 1 h at 37°C to allow CLR internalization (black squares). Results depict the average ± SE of a minimum of 5000 events. Results are representative of three independent experiments.

Dendritic cell maturation is generally thought to reduce antigen uptake, while increasing the T-cell stimulatory capacity (38). Down-regulation of CLR expression upon maturation (39) could contribute to this decreased antigen uptake function. Therefore, we tested CLR expression at different time-points after LPS-induced DC maturation and simultaneously evaluated CLR-mediated antigen internalization in these DCs. A decreased membrane expression was observed for all CLRs after overnight incubation with LPS (Figures 3A,C,E,G, white squares), whereby MGL expression already declined 1 h after LPS addition. Nevertheless, residual, yet significant, expression of all four CLRs was observed on mature DCs, suggesting that also in mature DCs, CLRs could facilitate antigen uptake. To test this hypothesis, we investigated the internalization capacity of the CLRs after the addition of LPS. Strikingly, the internalization scores and thus the internalization rates of all tested CLRs remained constant after the addition of LPS (Figures 3B,D,F,H, black squares). However, recycling of CLRs appeared to be affected in mature DCs. Although the recycling capacity of MR and DCIR only slightly decreased in mature DCs (Figures 3E,G), MGL was unable to recycle in mature DCs (Figure 3C, compare the black and white squares in the no LPS and o/n LPS conditions). This decline could already be observed 1 h after the addition of LPS. These results indicate that in spite of the reduced cell membrane expression, DC-SIGN, MR, and DCIR are still able to endocytose antigens in mature DCs. In addition, MGL was not able to recycle back to the cell membrane in mature DCs, even though its internalization capacity remained intact.

### Internalization characteristics of CLRs on other APCs

**Figure 4:**
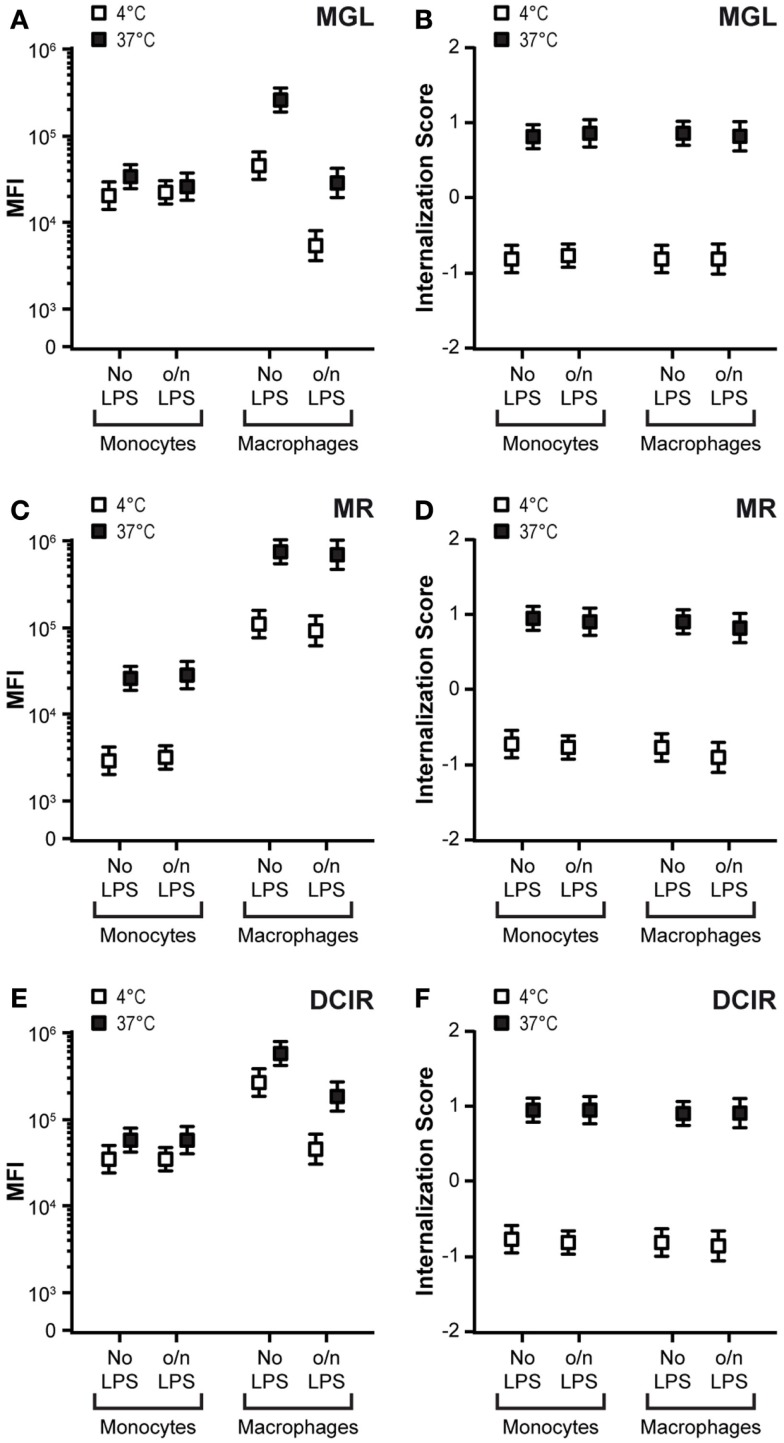
**Differences in recycling capacity on distinct APCs**. **(A,C,E)** Monocytes and macrophages were stimulated o/n with 10 ng/ml LPS. Cell membrane expression and recycling capacity were tested by CLR staining at 4°C (white squares) and subsequent recycling at 37°C (black squares). An increased median fluorescent intensity (MFI) at 37°C indicates receptor recycling. Results depict the average ± SE of a minimum of 5000 events. **(B,D,F)** Internalization scores of CLRs are given for monocytes and macrophages before and after LPS stimulation. Membrane-expressed CLRs were targeted with CLR-specific antibodies (4°C/white squares) and internalization was allowed for 1 h after removal of unbound antibodies (37°C/black squares). Results depict the average ± SE of a minimum of 5000 events. Results are representative of three independent experiments.

Mannose receptor, MGL, and DCIR are also expressed on other APCs such as macrophages and monocytes. Therefore, we investigated the antigen uptake capacity of these CLRs on unstimulated and LPS-triggered monocytes and macrophages. While CLR expression on monocytes was unaffected by LPS stimulation, macrophages displayed a clear decrease in DCIR and MGL expression and identical MR levels (Figures 4A,C,E). Strikingly, MGL and DCIR were practically unable to recycle in monocytes, while a substantial recycling capacity of MGL was present in macrophages (Figure 4A). LPS did not affect the recycling capacity of MGL, in contrast to DCs (compare Figure 4A with Figure 3C). The recycling capacity of DCIR was slightly reduced, but still significantly present (Figure 4C) and MR had a comparable recycling capacity in all cell types tested. In contrast, for all CLRs tested, internalization scores were similar in the different APC subsets (Figures 4B,D,F). These data suggest that CLRs maintain the ability to internalize their ligands independently of the cell type on which they are expressed, however the uptake of large amounts of antigens is prohibited for MGL on mature DCs and MGL and DCIR on monocytes, since their recycling capacity is lost in these cells.

### CLR internalization rates are not affected by simultaneous triggering of other CLRs

**Figure 5:**
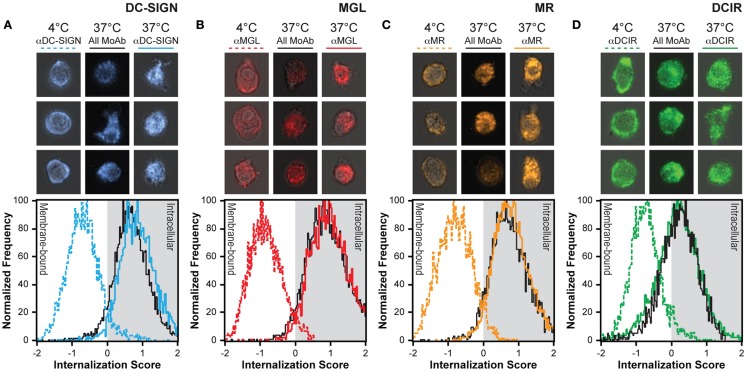
**Internalization is not affected by simultaneously triggering multiple CLRs**. **(A–D)** CLRs were allowed to internalize for 1 h in the presence (all MoAb) or absence (αCLR) of unlabeled antibodies for the other three receptors. The colored lines indicate the internalization score of a single CLR, while the black line depicts the internalization score of the indicated receptor upon combined CLR triggering. Three representative images of all conditions are given above the internalization scores. Results are representative of three independent experiments.

Although MR, DC-SIGN, and DCIR share recognition of certain mannose/fucose-containing glycans, MGL, specifically interacts with terminal GalNAc moieties. Pathogens and self-proteins are generally covered with a large variety of glycan structures, indicating that some antigens could carry ligands for more than one CLR. Therefore, simultaneous triggering of multiple CLRs is likely to occur and could potentially affect their individual internalization capacities. We next tested the ability of each of the four CLRs to internalize their ligand in the presence of specific antibodies to one or all other CLRs. However, none of the CLRs showed a reduced internalization capacity in the presence of the simultaneous stimulation of one or all other CLRs (Table 1 and Figure 5, respectively), indicating that CLR internalization is an independent process.

**Table 1:** **Simultaneous triggering of two CLRs does not affect their internalization**.

		Shift in internalization score			
		DC-SIGN	MGL	MR	DCIR
Simultaneous CLR triggering	DC-SIGN		2.14 ± 0.05	2.03 ± 0.09	1.25 ± 0.16
	MGL	2.05 ± 0.15		2.09 ± 0.12	1.35 ± 0.11
	MR	2.12 ± 0.13	2.28 ± 0.16		1.29 ± 0.17
	DCIR	2.18 ± 0.17	2.17 ± 0.11	2.13 ± 0.13	

*CLRs were simultaneously triggered in combinations of two and the internalization of each of the CLRs was addressed by imaging flow cytometry. Internalization scores ± SD are given for the different combinations. Results are representative of three independent experiments*.

### DCIR follows a distinct intracellular routing

**Figure 6:**
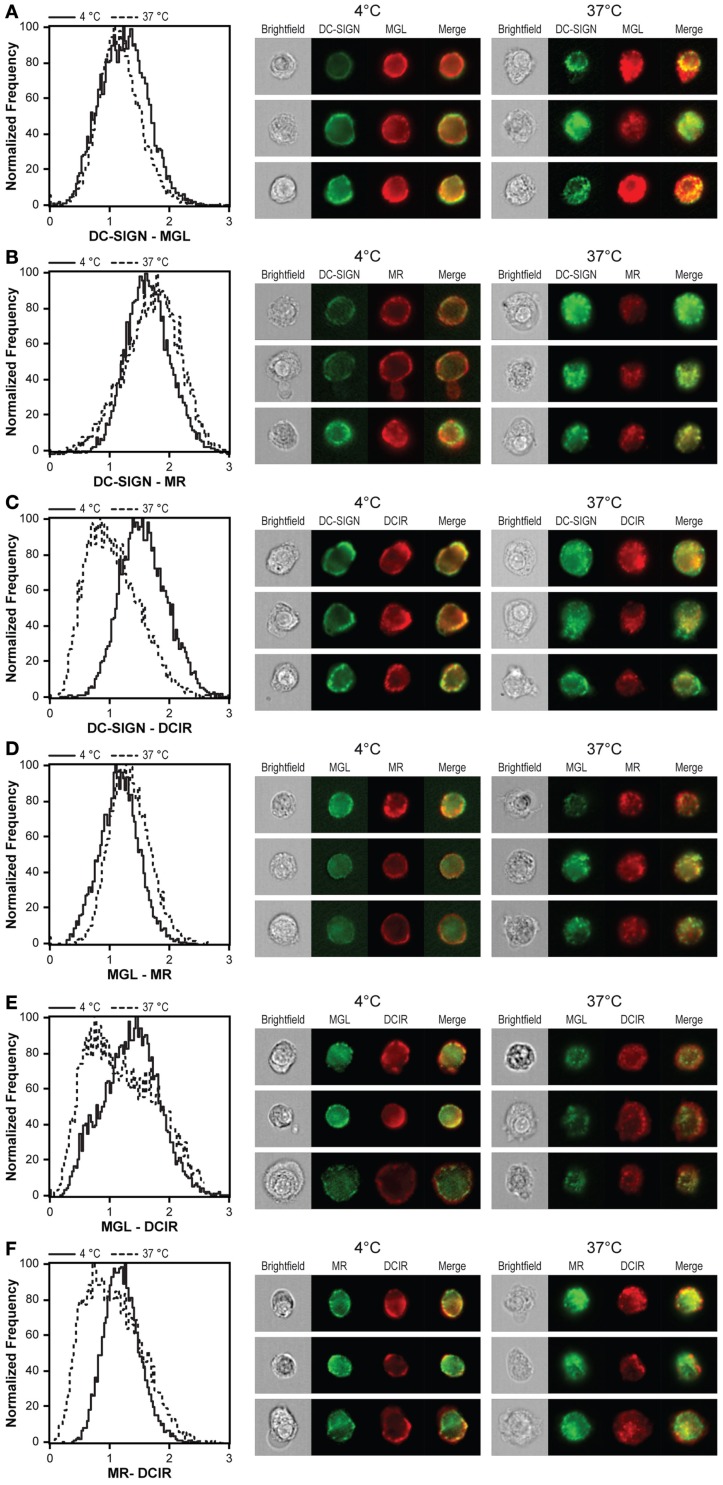
**DCIR follows a distinct internalization route compared to other CLR**. DCs were stained at 4°C with CLR-specific antibodies, washed, and incubated for 1 h at 37°C to allow CLR internalization. Co-localization scores of DC-SIGN and MGL **(A)**, DC-SIGN and MR **(B)**, DC-SIGN and DCIR **(C)**, MGL and MR **(D)**, MGL and DCIR **(E)**, and MR and DCIR **(F)** were calculated for both conditions and are depicted by the histograms on the left. A high co-localization score at 4°C indicates co-expression of CLRs on the cell membrane. A high co-localization score at 37°C indicates the presence of both lectins in the same intracellular compartment. Three representative images of all conditions are given next to the co-localization scores. Results are representative of three independent experiments.

Besides a possible effect on the basic internalization capacity, simultaneous triggering of CLRs could also affect their intracellular routing. Endo-lysosomal routing of antigens has been described for all four CLRs, indicating the existence of a common intracellular routing pathway and co-localization of different CLR ligands after internalization. We addressed this issue by calculating co-localization scores after triggering the internalization of all four CLRs simultaneously. Co-localization scores for DC-SIGN, MGL, and MR were similar at 4 and 37°C (Figures 6A,B,D), suggesting that internalization of these three CLRs proceeds from the plasma membrane to the same intracellular compartments. Strikingly, although DCIR showed co-localization with all CLRs at the plasma membrane (visualized by staining at 4°C), this co-localization was lost upon incubation at 37°C (Figures 6C,E,F). Together, these results suggest that DCIR follows a distinct intracellular routing than DC-SIGN, MGL, and MR.

**Figure 7:**
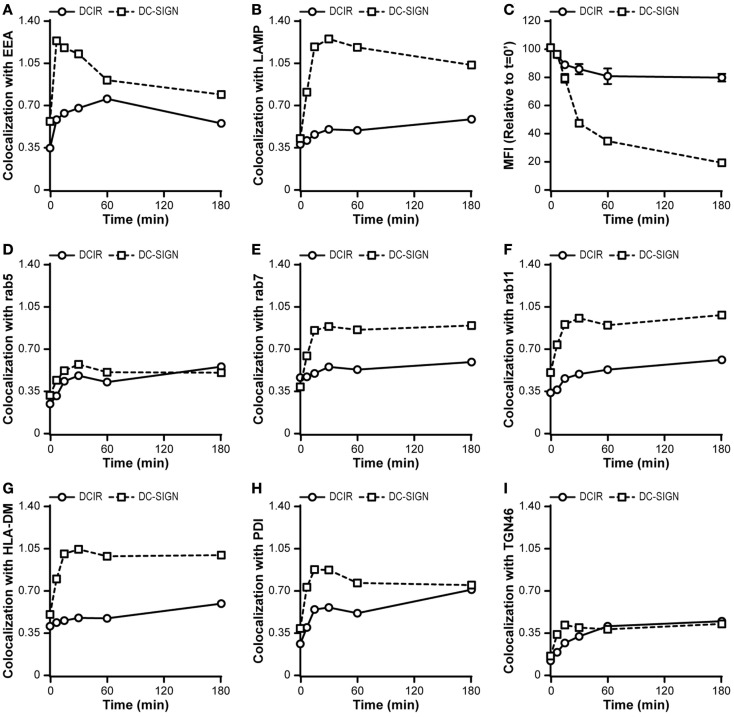
**DCIR follows an undefined internalization pathway**. **(A,B,D–I)** Membrane-expressed CLRs were stained at 4°C with CLR-specific antibodies and after washing excess antibodies away, the cells were transferred to 37°C for indicated times. Internalization of receptors was followed in time and co-localization scores with different intracellular routing markers were calculated. Markers used: the early endosomes (Rab5), late endosomes (Rab7), recycling endosomes (Rab11), MHC class II-loading compartments (HLA-DM), endoplasmic reticulum (PDI), and the trans Golgi network (TGN46) **(C)**. The degradation of internalized antibodies was assessed by measuring the decay of the fluorescent intensity corresponding to the fluorochrome conjugated to the antibody. The median fluorescent intensity (MFI) relative to time-point 0 is depicted. Results are representative of three independent experiments.

Therefore, we further dissected the internalization pathway of DCIR and compared this to the routing of antigens by DC-SIGN. In contrast to DC-SIGN, which rapidly routed to early endosomes and subsequently to lysosomes, DCIR showed a delayed co-localization with endosomes and only marginal co-localization with lysosomes (Figures 7A,B). The lack of lysosomal co-localization of DCIR correlated with a diminished loss of fluorescence of the DCIR antibody, indicating a decreased lysosomal degradation (Figure 7C). Within 3 h after internalization, the DC-SIGN-related fluorescence decayed to only 20% of the initial fluorescent signal, suggesting that lysosomal degradation occurred quickly via this route (Figure 7C). Based on the lack in co-localization of DCIR with the lysosomes, we hypothesized that DCIR-internalized ligands travel to a different intracellular compartment. To test this hypothesis, we measured the co-localization of DCIR with several markers of other intracellular vesicles. Strikingly, a poor co-localization was found with all compartments tested, including the early and late endosomes (Rab5 and Rab7), recycling endosomes (Rab11), MHC class II-loading compartments (HLA-DM), endoplasmic reticulum (PDI), and the Golgi (TGN46; Figures 7D–I). These data suggest that DCIR targets antigens to an as yet undefined early endosomal compartment.

### Combined triggering of DCIR affects the internalization route of DC-SIGN

**Figure 8:**
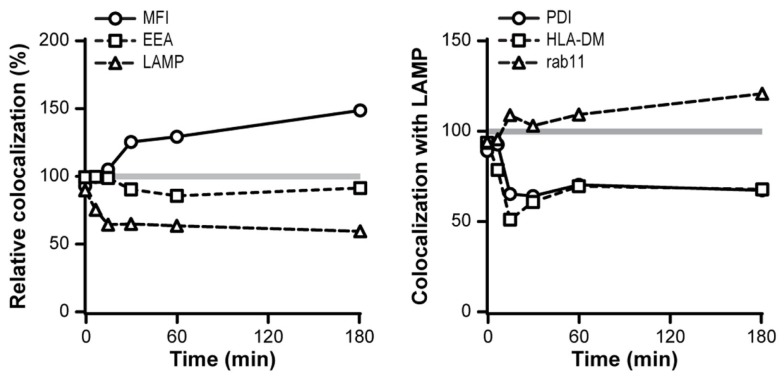
**DCIR triggering affects routing of DC-SIGN ligands**. Co-localization scores of DC-SIGN with indicated intracellular compartments were measured over time in combination with DCIR triggering. Co-localization in the absence of DCIR triggering was set to 100%. Markers used: endosomes (EEA-1), lysosomes (LAMP), recycling endosomes (Rab11), MHC class II-loading compartments (HLA-DM), and endoplasmic reticulum (PDI). The degradation of internalized antibodies was detected by measuring the fluorescent intensity of the AF405-labeled-DC-SIGN antibodies. Results are representative of three independent experiments.

Since DCIR and DC-SIGN have an overlapping glycan specificity and share binding to certain ligands (19), we investigated whether simultaneous triggering of DC-SIGN and DCIR would affect their intracellular routing. DC-SIGN triggering did not influence the intracellular routing of DCIR, which showed a similar low co-localization with known compartments in the presence of DC-SIGN stimulation (data not shown). However, the intracellular routing of DC-SIGN was affected upon simultaneous DCIR triggering, showing a decreased co-localization with lysosomes and a diminished decay in DC-SIGN-associated fluorescence (Figure 8). In addition, the co-localization scores of DC-SIGN with PDI (ER) and HLA-DM (MHC-II-loading compartments) were decreased, suggesting a reduced antigen presentation to T cells of DC-SIGN and DCIR-co-binding ligands. In contrast, co-localization of DC-SIGN with rab11 was slightly increased (Figure 8).

### Ligands endocytosed by DCIR are poorly presented to T cells

**Figure 9:**
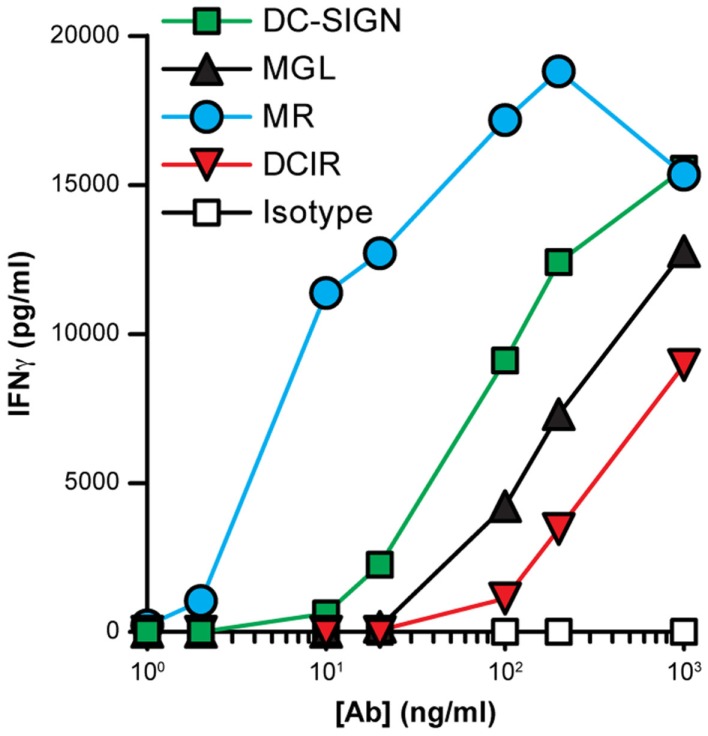
**Stimulation of CD4^+^ T cells after CLR-mediated internalization**. Immature DCs were incubated for 2 h with serial dilutions of CLR-specific or isotype control antibodies (all IgG1). Subsequent activation of an IgG1-specific CD4^+^ T-cell clone was analyzed by measuring IFNγ secretion by ELISA. Results are shown as the average of triplicate measurements and are representative of three independent experiments.

Since the routing of DCIR-internalized ligands was relatively slow and differed greatly from that of the other CLRs, we investigated whether DCIR was able to deliver antigens for MHC class II presentation to CD4^+^ T cells. DCs were incubated for 2 h with CLR-specific antibodies, after which a T-cell clone specific for an IgG_1_ derived-peptide was added. After 2 days, supernatant was taken from the co-cultures and IFNγ was measured as marker for T-cell activation. The most efficient antigen presentation was seen for antibodies targeting the MR, whereas the DCIR-binding antibodies clearly had the least ability to stimulate CD4^+^ T cells (Figure 9). Together, these results indicate an inferior function of DCIR in targeting antigens for presentation to CD4^+^ T cells.

## Discussion

Mannose receptor, DC-SIGN, MGL, and DCIR are four CLRs expressed by DCs of which their glycan specificity has been well-characterized. Although their role in antigen presentation has been investigated in detail, the actual antigen uptake and routing in immature and mature APCs has never been thoroughly investigated. Furthermore, most of the research is focused on the internalization of one single CLR, while many antigens or pathogens display several different glycan structures and could therefore interact with multiple CLRs at a time. To address these questions, we made use of imaging flow cytometry, which allows un-biased statistical analysis of morphological data in large cell populations. Our results have revealed differences in the recycling capacity of the four CLRs tested, which for MGL and DCIR was also dependent on the APC studied. Furthermore, DCIR exhibited a reduced internalization capacity as well as a distinct intracellular routing compared to the other CLRs. Lastly, simultaneous DCIR triggering affected the intracellular routing of DC-SIGN ligands, whereas no effects on the basal internalization were observed if all four CLRs were triggered simultaneously.

The intracellular domains of CLRs contain different motifs that mediate receptor-mediated endocytosis. DC-SIGN contains three such internalization motifs, namely a di-leucine, a tyrosine-based motif, and the triacidic cluster. We have previously shown that DC-SIGN-mediated internalization is mediated predominantly by the di-leucine motif (33). In contrast, the MGL and MR-mediated internalization is dependent on the tyrosine-based motif (7, 40, 41). The only putative internalization motif present in the cytoplasmic domain of DCIR is a tyrosine-based motif. The ability of MR, MGL, and DCIR to enhance antigen uptake by receptor recycling suggests that the tyrosine-based motif present in their intracellular tails might be involved in receptor recycling. Indeed, mutating tyrosine motifs decreased the recycling of CD22. Nevertheless, an additional glutamine residue in a membrane proximal motif was important for CD22 receptor recycling as well (42). However, more detailed mutational analysis would be required to fully elucidate the internalization mechanism of DCIR.

Receptor recycling could be the result of intrinsic relocalization of CLRs from intracellular pools to the cell membrane, or caused by the release of internalized antigens in the endocytic pathway, while the antigen uptake receptor returns to the cell surface. Based on our experiments, we cannot discriminate between these two forms of recycling for MR, MGL, and DCIR, since these receptors all contain an intracellular pool. The intracellular pool of MR has already been reported to be redistributed to the plasma membrane after MR triggering, resulting in an enhanced antigen uptake (31, 32). We here demonstrate that MGL and DCIR have a comparable distribution as the MR, which could contribute to the observed enhancement of antigen uptake. Conversely, recycling of receptors that originate from the cell membrane has been reported for DEC-205 and MR (43). Receptor recycling for DEC-205 is dependent on a triacidic cluster in its cytoplasmic domain. However, although a similar triacidic cluster is present in DC-SIGN, we did not observe any recycling of DC-SIGN. Therefore, DC-SIGN is the only CLR tested that does not function as recycling receptor on DCs.

Nonetheless, DC-SIGN-internalized ligands have been frequently reported as potent T-cell stimulators (2, 4, 6) and we here show that they appear to be even more effective in stimulating CD4^+^ T-cell activation than MGL and DCIR-binding ligands (Figure 9). This may be explained by either the more optimal routing of DC-SIGN-internalized ligands to MHC-II-loading compartments or by a positive effect of DC-SIGN signaling on T-cell stimulation. The recycling capacity of the other CLRs could, in contrast, contribute to a more efficient uptake of pathogens for their elimination. The expression of DCIR and MGL on macrophages further supports such a function.

Dendritic cell maturation induces a quick loss of the recycling capacity of MGL. Together with the decreased expression of CLRs on the plasma membrane, this could hypothetically contribute to a reduced CLR-mediated antigen uptake function in mature DCs (38). In addition, the overall endocytic capacity of mature DCs is decreased as compared to unstimulated DCs (44). Strikingly, the internalization capacity per CLR molecule was not affected by maturation and no effect on MR and DCIR recycling was found. A similar internalization capacity for DEC-205 on mature DCs has already been demonstrated (45), however expression of this receptor is up regulated after DC maturation, suggesting a different function of this receptor on mature DCs compared to the CLRs tested here. These results indicate that, for the four tested CLRs in this study, CLR ligands are taken up less efficiently by mature DCs, only due to a lower membrane expression.

Since many glycosylated antigens express multiple glycan structures, it is a likely possibility that one antigen can simultaneously interact with multiple CLRs. We here investigated the effect of simultaneous triggering of various CLRs on their antigen internalization and routing. No differences in internalization capacity were observed, suggesting that in the presence of abundant antigen, internalization of the antigen will be mediated by all CLRs interacting with the ligand. However, when only limited amounts of antigen are available, receptor affinity may determine which CLR dominates in mediating antigen uptake.

Here, we show that the routing of MR, MGL, or DC-SIGN is rather similar, since the comparative co-localization score of reciprocal CLRs remained constant during the internalization process. In contrast, DCIR appeared to be less prone to mediate internalization and followed a completely different intracellular pathway. One interesting possibility is that DCIR elicits ITIM-dependent signaling (11, 14, 46), which modulates the routing of ligands internalized via an alternative receptor. This might explain the effects observed on DC-SIGN routing upon simultaneous triggering of DCIR and may be related to the DCIR-mediated increased HIV infectivity previously described (47).

In addition, MR, MGL, or DC-SIGN have also been described to signal upon receptor engagement (17, 48). Both DC-SIGN and MGL triggering modulate TLR signaling to enhance IL-10 production (29, 48–50). Therefore, the simultaneous engagement of multiple CLRs could initiate signaling responses that potentially affect the routing of other CLRs, as we here describe for DCIR and DC-SIGN.

In order to compare the individual contribution of the different receptors, we used CLR-specific antibodies with, presumably, comparable binding affinities. However, the affinity constants of the natural ligands, glycans, are often several orders of magnitude higher than antibodies and may differ dramatically amongst receptors, whereby for instance glycan avidity may have a substantial effect on pathogen recognition (51). Furthermore, all our experiments were conducted using monocyte-derived DCs. However, primary DCs generally co-express multiple CLR receptors, suggesting CLRs may influence each other’s internalization and routing also *in vivo*. Thus, factors other than signaling or subcellular interactions may also be important in determining the outcome of immune responses when pathogens interact with multiple CLRs.

In conclusion, we here demonstrate that MGL and DCIR behave as recycling receptors on DCs, as has previously been reported for MR (31, 32). However, while MR behaves as a recycling receptor in different APC subsets, MGL and DCIR have a compromised recycling capacity in monocytes and macrophages. Neither maturation nor simultaneous triggering affects the internalization capacity of the CLRs investigated, however, the routing of DC-SIGN was compromised upon concomitant DCIR triggering. In addition, DCIR routes to an as yet unidentified endosomal compartment distinct from DC-SIGN, MR, or MGL, and DCIR-binding ligands seem to have a decreased capacity to stimulate CD4^+^ T cells. Together, our data contribute to a better understanding of CLR biology on DCs and will aid the design of targeting strategies for DC-based immunotherapies against cancer and infectious diseases.

## Conflict of Interest Statement

The authors declare that the research was conducted in the absence of any commercial or financial relationships that could be construed as a potential conflict of interest.

## Abbreviations

AF, alexa fluor; APC, antigen-presenting cell; BSA, bovine serum albumin; CLR, C-type lectin; DAMP, danger-associated molecular pattern; DC, dendritic cell; DC-SIGN, DC-specific intercellular adhesion molecule-3-grabbing non-integrin; DCIR, dendritic cell immunoreceptor; LPS, lipopolysaccharide; MGL, macrophage galactose-type lectin; MHC, major histocompatibility complex; MR, mannose receptor; PAMP, pathogen-associated molecular pattern; PBMC, peripheral blood mononuclear cell; PFA, paraformaldehyde; TLR, toll-like receptor.
